# Effectiveness of deep brain stimulation in Parkinson’s disease treatment with Single-center experience in Pakistan

**DOI:** 10.12669/pjms.39.4.7680

**Published:** 2023

**Authors:** Aurangzeb Kalhoro, Abdul Sattar M. Hashim

**Affiliations:** 1 Dr. Aurangzeb Kalhoro, F.C.P.S(Neurosurgery), F.A.C.S, M.B.A. Consultant Neurosurgeon, Neuro Spinal and Cancer Care Institute, Karachi, Pakistan; 2 Prof. Dr. Abdul Sattar M. Hashim, MD, Ph.D. Neurosurgery, Ex. Professor, JPMC, Karachi, Medical Director, Neuro Spinal and Cancer Care Institute, Karachi, Pakistan

**Keywords:** Parkinsonian disease, Deep brain stimulation, Levodopa, Tremor, Motor fluctuation

## Abstract

**Objective:**

To assess the effectiveness and accuracy of deep brain stimulation in Parkinsonian Disease (PD).

**Methods:**

This study was a descriptive prospective study, and patients were treated at Neurospinal and Cancer Care Institute Karachi, from February 1, 2016, to June 30, 2020. We had 21 cases of parkinsonian disease. Inclusion criteria was Idiopathic Parkinson’s disease, marked motor fluctuations against the response to dopaminergic therapy, UPDRS-III scores, which is 30 or higher, with a duration of disease of five years or longer, developing dyskinesia while the exclusion criteria was patient with known comorbid or active psychiatric disease

**Results:**

Mean age of patient was 64 years. The standard deviation was 1.11697. The male patients’ mean, median and mode had a standard deviation of 0.3. For the duration of disease, the mean was 1.4, the median 1 (5-6 years) and mode one. The standard deviation was 0.51177. The primary symptoms’ mean was 2.2857, the median was 2.0, and the mode was two (tremor). The mean on medication (age) was 2 (45-49), and the median and mode were the same.

**Conclusion:**

Deep brain stimulation **(**DBS) is an effective treatment option for a carefully selected patient. DBS improves tremors, dyskinesias, rigidity, motor fluctuations and bradykinesia. DBS is unlikely to benefit Autonomic dysfunction, cognitive disorders, hypophonia, and postural instability. Although it is an expensive treatment compared to lesioning or gamma knife, it is reversible.

## INTRODUCTION

The prevalence of Parkinson’s disease (PD) is around 1% of the population above the age of sixty, and the prevalence is directly proportional to increasing age for men and women.^1^ Pathophysiologically observed that the degeneration of dopaminergic nigrostriatal neurons with Lewy bodies is regarded as the main neuropathological correlate of motor impairment in Parkinson’s disease.^2^

Parkinson’s disease (PD) is considered the second most common brain neurodegenerative disorder after disorder as Alzheimer’s disease; it presents with motor symptoms that includes bradykinesia, resting tremor, postural problems and rigidity while other non-motor symptoms include autonomic manifestations (bowel problems such as sexual dysfunction, constipation and urinary complaints) neuropsychiatric problems (psychosis, anxiety, cognitive impairment, depression, compulsive disorders and apathy) and some sensory issues.^3^ In medication Levodopa up to date is considered one of the most useful medications to suppress signs and symptoms of PD, as time passes, long-term use results in Levodopa-induced dyskinesia.^4^

Multiple surgical and noninvasive options, such as stereotactic MR-guided focused ultrasound thalamotomy and pallidotomy for Parkinson’s disease area, are available;^5^ plus the gamma knife radiosurgery technique is an option to treat PD, which is a noninvasive method.^6^ In recent advancement, deep brain stimulation (DBS) is useful in controlling the motor symptoms in Parkinson’s disease (PD), which overall improve the quality of life of PD patients.^7^ Subthalamic nucleus stimulation by DBS (STN-DBS) is also an effective therapy against medication-refractory motor symptoms in patients with Parkinson’s disease.^8^ Another nucleus used for stimulation is globus pallidus internus in deep brain stimulation (GPi-DBS), depending on variation of signs and symptoms at presentation. At the same time, the outcome is measured by the scoring, Unified Parkinson’s Disease Rating Scale (UPDRS) preoperative and postoperative status.^9^

Deep brain stimulation (DBS) applies the deep brain structures, resulting in an effective treatment option for some neurological and psychological disorders. Other treatment options, which are conventional stereotactic surgery, such as thalamotomy and pallidotomy, helpful in making lesions in the globus pallidus and thalamus, and in comparison, are permanent, went through a renaissance. DBS is also applied for epilepsy treatment, treatment of pain, neuropsychiatric disorders, which are essential (resting tremor), obsessive-compulsive disorders, Tourette’s syndrome and depression.^10^

In the sub-continent, we have seen very limited studies based on intervention or surgical outcomes based on parkinsonian disease; most of them are treated by medication, while this study is one of the initial benchmark research on deep brain stimulation, which can lay a road map of future studies in Pakistan.

## METHODS

The study was conducted at the Neuro-Spinal & Cancer Care Institute, Karachi, it was a prospective cross-sectional study, and it was non-probability consecutive sampling. The hospital’s ethical committee approved the written permission from patients involved in the study (Ref No.: IRB#7412/12). The duration of the study was from 01/02/2016 to 30/6/2020. A comprehensive history with PD duration of more than five years, neurological examination and imaging magnetic resonance imaging brain. We had twenty-one patients, 19 male and two female patients with idiopathic PD undergoing Deep brain stimulation of the GPi / STN. Inclusion criteria Idiopathic Parkinson’s disease, patients show fluctuations related to motor function in response to the dopaminergic medication, UPDRS-III score of 30 or higher, duration of the disease more than five years or longer, developing dyskinesia and exclusion criteria patient with multiple comorbid, active psychiatric disease, coagulopathy, trauma, post brain surgery tremor.

Patients who went under the procedure were based on a one-stage electrode placement and stimulator implantation stimulated after one week. Unified Parkinson’s Disease Rating Scale III (UPDRS III) was utilized as an outcome measuring parameter primarily for motor function; scores had many components such as axial symptoms, limb bradykinesia, gait posture, stability and overall bradykinesia, another system based on scoring from 0-5 as if it gets higher, it may result in disability, Hoehn and Yahr disability scale.

**Fig.1:**
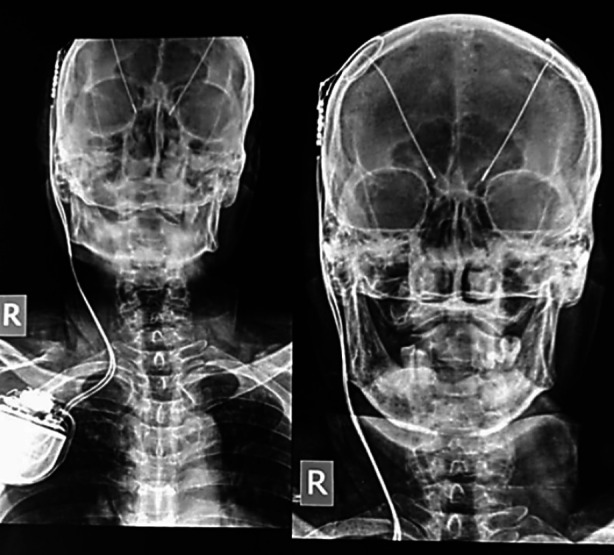
Showing post DBS x-ray. We can appreciate leads and Internal pulse generator (with patient’s permission).

Follow-up was at three and six months. The assessment, based on the evaluation of cognitive and motor function, was conducted by neurologists postoperatively to establish fine control of symptoms. The management included an adjustment of pharmacologic therapy and DBS parameter setting. The internal pulse generator is shown in the X-ray Fig.1 (picture added with permission).

## RESULTS

Our study’s mean age was 64, and the standard deviation was 1.11697. The mean, median and mode for a male were with a standard deviation of 0.3. For the duration of suffering, the mean was 1.4, the median 1 (5-6 years) and mode 1. The standard deviation was 0.51177; for primary symptoms, the mean was 2.2857, the median was 2.0, and the mode was 2 (tremor). The mean on medication (age) was 2 (45-49), and the median and mode were the same. For complications, the reported mean was 1.8571, the median was two, and the mode was two (no complications); the standard deviation was .35857. For pre-op UPDRS, the mean was 2.7619, median 2.0, and mode 2.0, and the standard deviation was 0.99523. For UPDRS DBS, the mean was 1.6667, the median was one and, the mode was also one, the standard deviation was .91287. for nuclei, the mean was 1.3333, the median was one, and the mode was also 1 (STN). The standard deviation was .48305.

The mean of the preoperative CGI score was 2.3333, the median was 2.0, the mode was 2.0, and the standard deviation was .57735. The mean postoperative CGI score was 2.4762, the median was 2.0, and the mode was 2.0 (2). the standard deviation was .81358. The mean of preoperative Hoehn & Yahr is 1.6667, median 2.0 and mode 2.0. the standard deviation was .6582. The mean of postoperative Hoehn & Yahr was 1.5714, median 2.0 and mode was also 2.0 (2). the standard deviation was .50709. Regarding frequency, 71.4% of the patients were 60 years and above, and more than 90% were male. All of them have been suffering for more than five years. 42.9% of patients reported tremors, and another 42.9% reported dyskinesia as the most frequent primary symptom. More than half (52.4%) of patients were of the 45-49 age group. as high as 85.7% of patients did not report any complications. Pre-op UPDRS had a frequency of 47.6% for the 65-69 age group, while 33.3% were for the 75-79 age group. UPDRS DBS was 61.9% for 25-29. the frequency of nuclei was 66.7% for STN. The frequency of preoperative CGI score 5 was found in 57.1% of patients and 6 for 38.1%. Table-I, II and III.

**Table-I:** Different Variables Shown (Statistics: Mean, Median, Mode and St. Deviation).

			Age		Gender	Duration of disease	Primary symptoms	On medication (age)	Complications reported
N		Valid	21		21	21	21	21	21
Mean			3.0476		1.0952	1.4762	2.2857	2.1905	1.8571
Median			3.0000		1.0000	1.0000	2.0000	2.0000	2.0000
Mode			4.00		1.00	1.00	2.00^a^	2.00	2.00
Std. Deviation			1.11697		.30079	.51177	.71714	1.03049	.35857

			*PRE-OP UPDRS*	*Post op UPDRS DBS*	*NUCLEI*	*PREOPERATIVE CGI SCORE*	*POST OPERATIVE CGI SCORE*	*PREOPERATIVE HOEHN & YAHR*	*POSTOPERATIVE HOEHN & YAHR*

N	Valid		21	21	21	21	21	21	21
Mean			2.7619	1.6667	1.3333	2.3333	2.4762	1.6667	1.5714
Median			2.000	1.000	1.000	2.000	2.000	2.000	2.000
Mode			2.0	1.0	1.0	2.0	2.0	2.0	2.00
Std. Deviation			.99523	.91287	.48305	.57735	.81358	.65828	.50709

a. The smallest value is shown, and multiple modes exist.

**Table-II:** Different variable with significant values

		Frequency	Percentage	Valid %	Cumulative %
Valid	50 – 54	3	14.30	14.30	14.30
	I55-5	3	14.30	14.300	28.60
	60-6	5	23.80	23.80	52.40
	65 And Above	10	47.6	47.6	100.0
	Total	21.0	100.00	100.00	

*Gender Frequency*					

		*Frequency*	*Percentage*	*Valid Percentage*	*Cumulative Percentage*

Valid	Male	19	90.5	90.5	90.5
	Female	2	9.5	9.5	100.0
	Total	21	100.0	100.0	

*Duration of Disease*					

		*Frequency*	*Percentage*	*Valid percentage*	*Cumulative Percentage*

Valid	5-6 Year	11	52.40	52.40	52.40
	7-8 Year	10.0	47.60	47.60	100.00
	Total	21.0	100.00	100.00	

*Sign & symptoms*		*Frequency*	*Percentage*	*Valid %*	*Cumulative %*

Valid	Motor fluctuations	3.0	14.3	14.30	14.30
	Tremor	9.00	42.9	42.90	57.10
	Dyskinesia	9.0	42.9	42.90	100.0
	Total	21.00	100.0	100.0	

*Frequency on Medication (Age)*					
		*Frequency*	*Percentage*	*Valid %*	*Cumulative %*

Valid	40-44	5.0	23.800	23.800	23.800
	45-49	11	52.4	52.4	76.2
	50-54	1	4.8	4.8	81.0
	55 and above	4	19.0	19.0	100.0
	Total	21.0	100.00	100.00	

*Frequency of Complications*					

		*Frequency*	*Percentage*	*Valid %*	*Cumulative %*

Valid	Yes	3	14.30	14.30	14.30
	No	18	85.70	85.70	100.0
	Total	21.0	100.00	100.00	

**Table-III:** Pre-Open UPDRS and Nuclei.

		Frequency	Percentage	Valid %	Cumulative %
Valid	60-64	1.0	4.80	4.80	4.80
	65-69	10	47.6	47.6	52.4
	70-74	3	14.3	14.3	66.7
	75-79	7	33.3	33.3	100.0
	Total	21.0	100.0	100.0	

*FREQUENCY OF UPDRS DBS*					

		*Frequency*	*Percentage*	*Valid %*	*Cumulative %*

Valid	25-29	13	61.90	61.90	61.90
	30-34	2	9.50	9.50	71.40
	35-39	6	28.60	28.60	100.00
	Total	21	100.0	100.0	

*FREQUENCY OF NUCLEI*					

		*Frequency*	*Percentage*	*Valid %*	*Cumulative %*

Valid	STN	14.0	66.70	66.70	66.70
	GPI	7.0	33.30	33.30	100.00
	Total	21.0	100.00	100.00	
	0000				

*The clinical global impressions scale (cgi)*					

		*Frequency*	*Percentage*	*Valid %*	*Cumulative %*

Valid	4	1	4.8	4.8	4.8
	5	12	57.1	57.1	61.9
	6	8	38.1	38.1	100.0
	Total	21.0	100.00	100.00	

*Frequency Of Postoperative Cgi Score*					

		*Frequency*	*Percentage*	*Valid %*	*Cumulative %*

Valid	1	2	9.5	9.5	9.5
	2	9	42.9	42.9	52.4
	3	8	38.1	38.1	90.5
	4	2	9.5	9.5	100.0
	Total	21.0	100.00	100.00	

*Frequency Of Preoperative Hoehn & Yahr*					

		*Frequency*	*Percentage*	*Valid %*	*Cumulative %*

Valid	TWO	9.0	42.90	42.90	42.90
	THREE	10.0	47.60	47.60	90.50
	FOUR	2.0	9.50	9.50	100.00eff
	Total	21.0	100.0	100.0	

*Frequency Of Postoperative Hoehn & Yahr*					

		*Frequency*	*Percentage*	*Valid %*	*Cumulative %*

Valid	ONE	9.0	42.90	42.90	42.90
	TWO	12	57.10	57.10	100.0
	Total	21	100.0	100.00	

Postoperative CGI score two was found in 42.9% of patients, while three was found in 38.1%. The preoperative HOEHN & YAHR of 3 was found in 47.6% of patients, while two were found in 42.9%. Postoperative HOEHN & YAHR 2 was found in 57.1% of patients, and one was found in 42.9% of patients.

## DISCUSSION

This is one of the selected studies on deep brain stimulation in the Asian sub-continent, especially Pakistan, with an acceptable result showing advancement in functional neurosurgery with deep brain stimulation helping patients affected by the side effects of parkinsonian medication.

One of the studies showed high mortality with other targets, while GPi was selected as the best target for severe dyskinesia. DBS may not affect the disease progress while patients should be selected precisely^11^ compared to our study, we did not have any mortality either with GPi or STN nucleus related to DBS the reason maybe the study is limited.

While in another recent study, they used functional images of an MRI brain for deep brain stimulation in patients for the subthalamic nucleus. The study also showed that DBS affects behavior-independent effects on the basal ganglia connectivity and the behavior-dependent modulatory effect^12^ while we used the Gama plan technique in our patients, which was easier in comparison.

In another study, it was shown that unilateral STN DBS had significantly improved the depression in a patient with PD, while it not only improved depression but also had refinement in the quality of patient life, as well as sleep quality^13^ compared to our study we did not have any patient with severe case of depression but the patient who had improved status and UPDRS score due to DBS had improved mood as compared to preoperative status but a review of the relevant literature, show that DBS patient does present with mood swings.

In another study, thirty patients for the parkinsonian study were taken, the outcome of which eight had negative, eight had a mix, and fourteen had a positive outcome; the study shows that all groups improved in motor function. Negative outcome patient expected reality result. On the other hand, the mixed-method approach had to examine a patient’s discrimination based on subjective elements of STN-DBS outcome, and parkinsonian disease patients should be screened thoroughly for other aspects such as apathy and depression before considering surgery.^14^

While a study also suggested that microelectrode recording is unnecessary to have good outcomes clinically in P.D patients undergoing STN DBS. This may help to design future perspectives.^15^ It is considered attainable to perform the deep brain stimulation procedure without using microelectrode recording (MER). However, microelectrode recording gives a valid choice to the team for utilizing a second trajectory which may improve the effectiveness of therapy, minimizing the side effects,^16^ even other articles show that microelectrode recording may or may not be utilized in the future as a procedure of choice, but its importance may persist in DBS^17^ while we did not perform microelectrode recording during DBS procedure and we had reasonable results.

One of the reports showed that moderate to severe complications for movement disorders is hydrocephalus due to deep brain stimulation, hydrocephalus may be related to the intraventricular hemorrhage postoperatively, which can be seen in some of the studies;^18^ however, during our operational cases related to Parkinson’s disease, we did not have any patients who had developed hydrocephalus post-procedure during follow up nor did any patient had hemorrhage.

While in another study, it was found that deep brain stimulation may have a low complication rate if performed under general, awake DBS patients have minimal post-surgical treatment-induced side effects while at the experienced center, DBS is performed under general anaesthesia;^19^ similarly, to this, we performed all our cases under general anesthesia with minimal post-surgical side effects, as mentioned in the results.

Individualized stimulation delivery is an advanced approach to deep brain stimulation. Usually, the two brain targets in treating Parkinson’s disease are an internal segment of the subthalamic nucleus (STN) and globus pallidus (GPi). In the present era, these two areas are highly focused on surgical decisions to implant Deep brain stimulation.^20^ In our study, 28.5% of patients had GPi, and 71.4% had STN.

Proper clinical trials were done in 1993, focusing on improving the non-damaging approach with the location, size, intensity and frequency of electric current and the shape of the stimulating current field. The trials were significantly reported as successful in enhancing motor functions. In addition, the least to no complication is reported by this approach to improve the advanced stage of Parkinson’s disease.^20^ In our study, 85.7% of the patients did not report any complications; only three had reported complications related to cognitive, rigidity and dysarthria, which were managed accordingly.

### Limitations:

The study has a limited number of patients as treatment is costly; the follow-up is kept short of giving our initial experience term to assess the initial effect of therapy that is to reduce medication and improve the status of the patient while in future long-term follow-up may be assessed.

## CONCLUSION

DBS is a method that can work effectively in treating Parkinson’s disease at an advanced stage. From this approach, very few patients reported complications due to comorbidity. Over the world, this approach is a priority for neurosurgeons to treat advanced-level Parkinson’s disease. The National and International neurological Association should look into this matter to make this technology affordable for middle to low-income groups. In addition, further awareness should be created about Parkinson’s disease and its complications affecting the quality of life.

### Authors’ Contribution:

**AK:** Conception or design of the work, or the acquisition, Statistical analysis, interpretation of data for the work.

**ASMH:** Drafting the work, working on the critically for important intellectual content and Final approval of manuscript.
